# Zinc Oxide Nanoparticles Prime a Protective Immune Response in *Galleria mellonella* to Defend Against *Candida albicans*

**DOI:** 10.3389/fmicb.2021.766138

**Published:** 2021-12-10

**Authors:** Mei-nian Xu, Li Li, Wen Pan, Huan-xin Zheng, Meng-lei Wang, Xiao-ming Peng, Si-qi Dai, Ying-mei Tang, Kang Zeng, Xiao-wen Huang

**Affiliations:** ^1^Department of Dermatology, Nanfang Hospital, Southern Medical University, Guangzhou, China; ^2^Division of Infectious Diseases, Rhode Island Hospital, Warren Alpert Medical School of Brown University, Providence, RI, United States

**Keywords:** zinc oxide nanoparticles, *Galleria mellonella*, *Candia albicans*, innate immunity, anti-infection

## Abstract

**Purpose:** Zinc oxide nanoparticles (ZnO-NPs) have exerted antimicrobial properties. However, there is insufficient evaluation regarding the *in vivo* antifungal activity of ZnO-NPs. This study aimed to investigate the efficacy and mechanism of ZnO-NPs in controlling *Candida albicans* in the invertebrate *Galleria mellonella*.

**Methods:** *Galleria mellonella* larvae were injected with different doses of ZnO-NPs to determine their *in vivo* toxicity. Non-toxic doses of ZnO-NPs were chosen for prophylactic injection in *G. mellonella* followed by *C. albicans* infection. Then the direct *in vitro* antifungal effect of ZnO-NPs against *C. albicans* was evaluated. In addition, the mode of action of ZnO-NPs was assessed in larvae through different assays: quantification of hemocyte density, morphology observation of hemocytes, characterization of hemocyte aggregation and phagocytosis, and measurement of hemolymph phenoloxidase (PO) activity.

**Results:** Zinc oxide nanoparticles were non-toxic to the larvae at relatively low concentrations (≤20 mg/kg). ZnO-NP pretreatment significantly prolonged the survival of *C. albicans-*infected larvae and decreased the fungal dissemination and burden in the *C. albicans-*infected larvae. This observation was more related to the activation of host defense rather than their fungicidal capacities. Specifically, ZnO-NP treatment increased hemocyte density, promoted hemocyte aggregation, enhanced hemocyte phagocytosis, and activated PO activity in larvae.

**Conclusion:** Prophylactic treatment with lower concentrations of ZnO-NPs protects *G. mellonella* from *C. albicans* infection. The innate immune response primed by ZnO-NPs may be part of the reason for the protective effects. This study provides new evidence of the capacity of ZnO-NPs in enhancing host immunity and predicts that ZnO-NPs will be attractive for further anti-infection applications.

## Introduction

*Candida albicans* is a prevalent opportunistic pathogen that can cause mucosal, cutaneous, and deep-seated organ infections in humans. *C. albicans* infection is the leading cause of systemic candidiasis, which is responsible for the high mortality among immunosuppressed or immunocompromised patients in hospitals (Gudlaugsson et al., 2003; Kullberg and Arendrup, 2016). Due to the limited antifungals against *C. albicans* and the continuous emergence of drug resistance, more effective and long-term antifungal materials are desperately needed (Salazar et al., 2020).

In recent years, metallic nanoparticles (NPs), such as silver, gold, copper, zinc, and iron NPs, have exhibited antimicrobial potentials (Staron and Dlugosz, 2021). NPs exert antimicrobial properties through multifaceted mechanisms, including, but not limited to, induction of reactive oxygen species (ROS), cell membrane damage, and interaction with biomolecules (Kim et al., 2009; Staron and Dlugosz, 2021). Among diverse types of NPs, silver nanoparticles (Ag-NPs) are most widely used as antimicrobials. However, the application of Ag-NPs has drawn public concerns due to the side effects, including toxicity, oxidative stress, and inflammatory response (Burns et al., 2021). Different from Ag-NPs, zinc oxide nanoparticles (ZnO-NPs) are safer to mammalian cells (Bondarenko et al., 2013; Efthimiou et al., 2021). Bondarenko et al. (2013) pointed out that the median lethal doses (LD50) of Ag-NPs and ZnO-NPs for mammalian cells were 11.3 and 43 mg/L, respectively. Moreover, ZnO-NPs possess several other advantages, such as chemical and thermal stability, high oxidation energy, and good antimicrobial properties, facilitating their wide application (Sharma et al., 2020). ZnO-NPs have a wide range of antifungal effects, including *C. albicans*, *Trichophyton*, *Microsporum canis*, *mentagrophytes*, *Aspergillus flavus*, *Sclerotinia homoeocarpa*, and *Fusarium oxysporum* (Sun et al., 2018). In addition, ZnO-NPs can increase the antifungal capacity of other materials, such as flax pulp (Abo Elgat et al., 2020). These studies suggest that ZnO-NPs have the potential to be an alternative to the classical antifungals. However, the current research mainly focused on the potential applications of NPs in agriculture (Hernandez-Melendez et al., 2018). Most antimicrobial data of ZnO-NPs were obtained from *in vitro* assays, stimulating the *in vivo* studies to elucidate their toxicity and antifungal activity.

Considering their specific characteristics, the invertebrate *Galleria mellonella* larvae have been widely used as an animal model for evaluating the virulence of pathogens and the efficacy of antimicrobial agents. For instance, larvae have a short lifespan, low cost, no ethical implications, and no requirement for special lab equipment (Cutuli et al., 2019). Most importantly, the innate immune response of larvae is much similar to that of vertebrates, such as identical phagocytic mechanisms, cell surface receptors, and cell signaling pathways (Cutuli et al., 2019; Tsai et al., 2020). In addition, preexposure to components of microbial cells, antifungals, or physical stress, primes larval immunity to defend against subsequent infection (Huang X.W. et al., 2020; Sheehan et al., 2020). Thus, *G. mellonella* presents highly functional similarities with aspects of the vertebrate innate immune memory (Netea et al., 2016). Furthermore, many human pathogenic microbes display comparable virulence in *G. mellonella* and mammals (Desbois and Coote, 2012). Therefore, *G. mellonella* is a feasible and straightforward alternative model for mammals. Recently, several studies have employed *G. mellonella* larvae as an animal model to screen nanomaterials (Moya-Anderico et al., 2021b). Although *Eskin* and his colleagues have reported the toxicity of ZnO-NPs in larval hemocyte counts, there is no report on the antifungal activity of ZnO-NPs in *G. mellonella* larvae (Eskin et al., 2019). Herein, this study aimed to evaluate the antifungal activity of ZnO-NPs by utilizing *G. mellonella* larva as an *in vivo* animal model and clarify the potential mechanism.

## Materials and Methods

### Preparation of Zinc Oxide Nanoparticle Suspensions

Zinc oxide nanoparticles were purchased from Sigma-Aldrich (St. Louis, MO, United States). The particles were dispersed in sterile phosphate-buffered saline (PBS, Thermo Scientific, Wilmington, DE, United States) at a concentration of 100 mg/ml as the stock solution. Then autoclaved stock solution was diluted in PBS to produce the working solutions at different concentrations. All working suspensions were ultrasonically dispersed for 15 min prior to experiments.

### Characterization of Zinc Oxide Nanoparticles

The surface morphology of ZnO-NPs was observed by scanning electron microscope (SEM; Zeiss Sigma 300, Oberkochen, Germany). The average particle size and shape of ZnO-NPs were further authenticated by a high-resolution transmission electron microscope (HR-TEM; FEI Tecnai G2 F20 S-TWIN, Hillsboro, OR, United States) at an accelerating voltage of 200 kV. The zeta potential of ZnO-NPs was measured using a Zetasizer Nano ZS (Nano-ZS; Malvern Instruments, Worcestershire, United Kingdom) at 25°C and analyzed by Zetasizer software.

### *Galleria mellonella* and *Candida albicans*

*Galleria mellonella* larvae were purchased from Wax Moth Breeding Inc., Tianjin, China. Larvae, weighing around 250 mg, were chosen for all experiments. Since food deprivation increases infection susceptibility of larvae (Junqueira, 2012), larvae were incubated without any food at 15°C for 24 h before use. Treated larvae were kept in Petri dishes and maintained in the dark at 37°C without any food source.

*Candida albicans* standard strain SC5314 was purchased from the American Type Culture Collection (ATCC, Manassas, VA, United States) and cultivated in yeast peptone dextrose (YPD) broth (Sangon Biotech, Shanghai, China) under 200 rpm at 37°C.

### Toxicity of Zinc Oxide Nanoparticles on *Galleria mellonella*

Each testing group contains 15 larvae. Before injection, the larvae were sterilized with 75% ethanol wipe. The larvae were injected with a Hamilton syringe (Sangon Biotech, Shanghai, China) *via* the last left proleg. Five microliters of ZnO-NP suspensions was administrated at concentrations of 0.1, 1, 10, 20, 40, and 80 mg/ml, which are equivalent to the dosages of 2, 20, 200, 400, 800, and 1,600 mg/kg, respectively, for a larva weighing 250 mg. The same volume of sterilized PBS was injected as the treatment control. After inoculation, the larvae were transferred to Petri dishes and maintained at 37°C for 8 days in the dark. The daily observation was performed to record larval mortality. Dead larvae are inert and usually turned black. Every experiment was thrice repeated. The toxicity of ZnO-NPs is represented by larval mortality.

### Infection Model of Zinc Oxide Nanoparticle-Pretreated *Galleria mellonella*

Each testing group contains 15 larvae. The non-toxic doses (0.2 and 2 mg/kg, 5 μl) of ZnO-NPs were chosen for prophylactic injection to ascertain the effects of ZnO-NPs on controlling *C. albicans* sublethal infection in *G. mellonella* larvae. Larvae pretreated with amphotericin B (4 mg/kg, 5 μl) and sterilized PBS were used as the positive and negative controls, respectively. Two hours later, the insects were challenged with a 70% lethal dose (LD70) of *C. albicans* as described previously (Huang X. et al., 2020, Huang X.W. et al., 2020). In brief, each pretreated larva was inoculated with 5 μl of *C. albicans* suspensions containing 1 × 10^5^ colony-forming unit (CFU) yeast cells *via* the last right proleg. After inoculation, larvae were transferred to Petri dishes and maintained at 37°C in the dark for an indicated time. Daily observation was conducted for recording larval mortality. The experiment was thrice repeated.

### Hemolymph Collection

Larvae were swabbed with 75% ethanol and positioned on a lid of Perish dish. The hemolymph was obtained by a syringe in the larval abdomen. Hemolymph that emerged from each larva was separately transferred into a microtube containing ice-cold insect physiological saline (IPS: 150 mM NaCl, 5 mM KCl, 0.1 M Tris–HCl, 10 mM EDTA, 30 mM sodium citrate, pH 6.9) (Procell, Wuhan, China) and 0.002% phenylthiourea (PTU; Leyan, Shanghai, China).

For PO activity assay, hemolymph was collected in the absence of IPS and PTU.

### Histopathological Analysis and Determination of Fungal Burden in *Galleria mellonella*

To evaluate the fungal morphology, distribution, and burden in ZnO-NP-pretreated larvae, the histopathological analyses of larvae were performed according to a previously described method (Spadari et al., 2019). Briefly, 48 h after infection, 15 larvae in each group were injected with 100 μl of 10% neutral-buffered formalin *via* the last left proleg and then fixed in formalin at 4°C overnight. After that, the whole larvae were sagittally dissected into two halves and fixed for another 48 h at 4°C. Next, samples were dehydrated with gradient ethanol, cleared with xylene, infiltrated with paraffin, embedded in paraffin, and subsequently sectioned at a thickness of 6 μm. The sections were stained with hematoxylin and eosin (H&E) and examined using a BX63 optical microscope (OLYMPUS, Tokyo, Japan).

The fungal burden in the hemolymph of the infected larvae was determined after inoculation with *C. albicans* for 3 and 9 h. Hemolymph was obtained from 15 replicates per group and was 10-fold diluted in PBS. Then, 100 μl of diluted hemolymph was spread to YPD solid medium containing 1% chloramphenicol and gentamicin. The agar plates were incubated at 37°C for 24 h. Colony counts were scored for measuring the fungal load.

### Determination of Direct Antifungal Effects of Zinc Oxide Nanoparticles Against *Candida albicans*

The direct antifungal activity of ZnO-NPs against *C. albicans* was confirmed by determining its minimum inhibitory concentration (MIC) using the broth microdilution methods following the guidance given by the Clinical and Laboratory Standards Institute (National Committee for Clinical Laboratory Standards, 2002). Briefly, 80 μl of YPD broth with various concentrations of ZnO-NPs was added to each well of a 96-well plate, and then 20 μl of yeast suspension containing 1 × 10^5^ CFU of *C. albicans* was added into each well. *C. albicans* incubated with amphotericin B (0.5 μg/ml) was used as the positive control. *Candida albicans* incubated in the absence of ZnO-NPs and amphotericin B was used as the negative control. After incubation at 37°C for 24 h, the growth of *C. albicans* was assessed by measuring optical density at 600 mm. The MIC was defined as the lowest concentration of ZnO-NPs at which no visible microbial growth was observed.

In addition, microbiological plating was conducted to evaluate the antifungal effects of ZnO-NPs. Briefly, 100 μl of PBS solution containing 1, 10, and 100 μg of ZnO-NPs was evenly spread on YPD plates containing 1% chloramphenicol and gentamicin. The same volume of PBS was coated as a control. Subsequently, 50 μl of yeast suspension with 60 CFU of *C. albicans* was applied to the coated plates followed by incubation at 37°C for 24 h. Colony counts and diameter were recorded for assessing the growth of *C. albicans*, indicating the antifungal activity of ZnO-NPs.

### Quantification and Morphological Observation of *Galleria mellonella* Hemocytes

For total hemocyte quantification and morphological observation, the hemolymph was collected from larva at the indicated time points after ZnO-NP pretreatment or *C. albicans* infection. Next, hemolymph from each larva was 10-fold diluted with ice-cold IPS containing 0.002% PTU in a 12-well plate. Twenty microliters of the diluted hemolymph was used for hemocyte counting immediately by an automated cell counter (Countstar, Shanghai, China). The remaining hemolymph was used for morphological observation using a BX51 optical microscope (Olympus, Tokyo, Japan).

### Scanning Electron Microscopy of Hemocytes

The microscopic morphology of hemocytes was assessed by SEM according to the method described previously (Kim et al., 2021). The ZnO-NP-pretreated larvae were challenged with *C. albicans* for 9 h. Then, hemolymph from 15 larvae in the same group was pooled into a 15-ml centrifuge tube containing IPS and 0.002% PTU. Hemocytes were sedimented by centrifugation for 200 × *g* at 4°C, following by washing thrice with PBS. Next, the samples were resuspended and fixed with 2.5% glutaraldehyde solution (Leagene Biotechnology, Beijing, China) for 24 h at 4°C. Then samples were dehydrated with increasing concentrations of ethanol (30, 50, 70, 80, 90, and 100%) and placed on an aluminum dish overnight to dry at room temperature. Subsequently, the samples were coated with gold and observed by SEM (Hitachi S-3000N, Tokyo, Japan).

### Phagocytosis Assay of Hemocyte

The phagocytosis by hemocytes was performed *in vivo* according to previous studies (Feldman et al., 2012; Wu et al., 2016). In brief, 15 larvae per group were inoculated with 5 μl of PBS or ZnO-NP suspensions (2 mg/kg) *via* the last left proleg. After incubation at 37°C for 2 h, each larva was challenged with fluorescein isothiocyanate isomer I (FITC; Solarbio, Beijing, China)-labeled *C. albicans* (1 × 10^5^ cells, 5 μl) and incubated at 37°C for 3 h in dark. For preparing FITC-labeled *C. albicans*, *C. albicans* suspensions (1 × 10^9^ cells/ml) were incubated with 0.1 mg/ml in a shaking incubator in the dark for 1 h and washed with PBS three times.

Subsequently, 50 μl of hemolymph was obtained from each larva and diluted in a 12-well plate containing 950 μl of IPS and 0.002% PTU. The plate was gently shaken followed by standing for 15 min at room temperature. The phagocytosis of *C. albicans* by hemocytes was visualized using an IX73 fluorescence microscope (Olympus, Tokyo, Japan).

### Phenoloxidase Activity Assay

Phenoloxidase (PO)-catalyzed melanization is a crucial innate defense response in *G. mellonella* larvae, displaying similarities to the complement system in vertebrates (Sheehan et al., 2020). The phenoloxidase activity in the hemolymph of larvae was measured at 2, 5, 8, and 11 h after dosing PBS or ZnO-NPs (2 mg/kg). Moreover, larvae receiving ZnO-NP pretreatment and *C. albicans* infection (1 × 10^5^ cells per larva) were used for phenoloxidase activity detection at 3, 6, and 9 h after infection. The phenoloxidase activity assay was conducted according to a previous report (Thomaz et al., 2020). Briefly, 2 μl of hemolymph sample was diluted in 18 μl of Tris buffer saline containing 5 mM CaCl_2_, followed by dilution with 180 μl of 2 mM L-DOPA (Solarbio, Beijing, China) in 50 mM sodium phosphate at pH 6.5. The enzyme activity was quantified by reading the absorbance at 490 nm over 90 min at 30-min intervals using a microplate reader (Bio-Rad, Hercules, CA, United States).

### Statistical Analysis

Fifteen larvae were used per group in one experiment, and each experiment was repeated in triplicate. Data were analyzed using the SPSS software 15.0 (SPSS Inc., Chicago, IL, United States). The survival curves of *G. mellonella* were plotted and examined using the Kaplan–Meier method. The differences in the survival curves were determined using the log-rank test. The hemocyte density, colony counts, and phenoloxidase activity level were shown as mean ± standard deviation (SD). The differences in the mean values between groups were assessed using Student’s *t*-test or one-way analysis of variance (ANOVA) methods. A *p*-value less than 0.05 in all replicate experiments was considered statistically significant.

## Results

### Characterization of Zinc Oxide Nanoparticles

**FIGURE 1:**
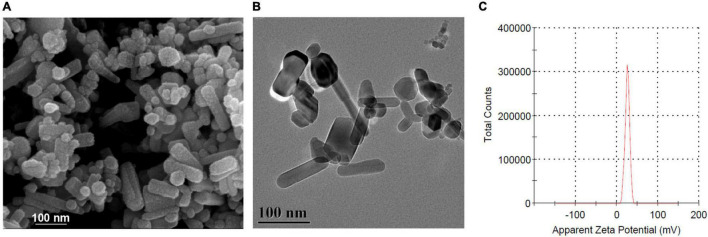
Characterization of zinc oxide nanoparticles (ZnO-NPs). **(A)** Scanning electron microscopy (SEM) and **(B)** high-resolution transmission electron microscope (HRTEM) showing the morphology of ZnO-NPs. Scale bar, 100 μm. **(C)** Zeta potential of ZnO-NPs.

Scanning electron microscope and HR-TEM were used to characterize the morphology of ZnO-NPs. As shown in Figures 1A,B, ZnO-NPs are nanorod shaped with an average size of 100 nm × 15 nm. Zeta potential, one of the fundamental parameters to evaluate the physical stability of nanosuspension, reflects the electric potential difference at the particle–liquid interface (Dananjaya et al., 2020). The zeta potential of ZnO-NPs was calculated as +25.9 mV (Figure 1C), inferring that ZnO-NPs exhibited good stability in water.

### Low Doses of Zinc Oxide Nanoparticles Were Safe to the *Galleria mellonella* Larvae

**FIGURE 2:**
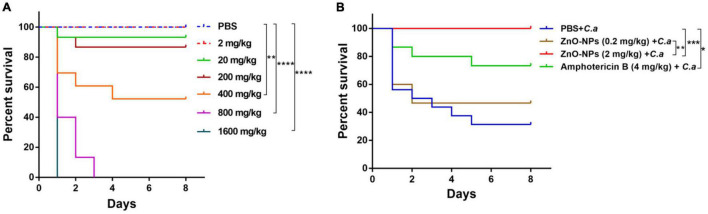
The toxicity of ZnO-NPs on *Galleria mellonella* and the efficacy of ZnO-NPs saving *G. mellonella* from *Candida albicans* infection. **(A)** Survival curves of *G. mellonella* larvae injected with various concentrations of ZnO-NPs. Larvae were injected with ZnO-NP suspensions at concentrations ranging from 2 to 1,600 mg/kg. Phosphate-buffered saline (PBS) was injected as the control. **(B)** Survival curves of *G. mellonella* receiving ZnO-NP pretreatment and *C. albicans* challenge. Larvae were pretreated with ZnO-NPs at non-toxic concentrations (0.2 and 2 mg/kg). Pretreatment with amphotericin B (4 mg/kg, 5 μl) and sterilized PBS was used as the positive and negative controls, respectively. Larvae were immunized with 1 × 10^5^ colony-forming units (CFU) of *C. albicans* 2 h postinoculation. Fifteen larvae were used per group in one experiment. The experiments were repeated in triplicate independently. Differences were determined by using the log-rank test. *p < 0.05; **p < 0.01; ***p < 0.001; ****p < 0.0001.

The *in vivo* toxicity of ZnO-NPs was assessed by utilizing *G. mellonella* larvae. We observed that the ZnO-NP treatment affected larval survival in a dose-dependent manner (Figure 2A). Specifically, the evident toxicity of ZnO-NPs on larvae was noticed at concentrations greater than 400 mg/kg. The LD50 and maximum LD (LD99) values of ZnO-NPs were 400 and 800 mg/kg, respectively. In addition, all larvae died at the second day after ZnO-NPs treatment at 1,600 mg/kg. As the injected dose of ZnO-NPs decreased, the larval survival rate increased. As illustrated in Figure 2A, treatments with 20 and 2 mg/kg of ZnO-NPs did not show any significant difference in the larval survival compared with PBS-treated control. Especially, no larva died after receiving 2 mg/kg of ZnO-NP treatment. These results indicated that low doses of ZnO-NPs were non-toxic to larvae and could be administrated in animal model research.

### Zinc Oxide Nanoparticle Pretreatment Improved the Survival of *Candida albicans*-Infected Larvae

The efficacy of ZnO-NPs defending against fungal infection was investigated *in G. mellonella* larvae. According to our previous research (Netea et al., 2016) and the *in vivo* toxicity results of ZnO-NPs on *G. mellonella* larvae (Figure 2A), ZnO-NPs at a non-toxic concentration of 2 mg/kg were applied for larval pretreatment in the following studies. Also, a relatively lower concentration (0.2 mg/kg) was performed to examine the dose-dependent bio-effects of ZnO-NPs. As shown in Figure 2B, the pretreated larvae were challenged with a 70% lethal dose (LD70) of *C. albicans* instead of a full lethal dose. After 8 days, 67% of the PBS-pretreated control larvae and 27% of amphotericin B-pretreated (4 mg/kg) larvae died of *C. albicans* infection. Meanwhile, after challenging with *C. albicans*, the ZnO-NP-pretreated larvae displayed significant viability compared with PBS-pretreated group. ZnO-NP pretreatment at a dose of 0.2 mg/kg reduced the larval mortality by 13% compared with PBS pretreatment (*p* > 0.05). All the infected larvae survived under pretreatment with ZnO-NPs at a dose of 2 mg/kg. These results indicated that ZnO-NPs were efficient in combating *C. albicans* at non-toxic doses.

### Zinc Oxide Nanoparticle Pretreatment Decreased the Load of *Candida albicans* in Larvae

**FIGURE 3:**
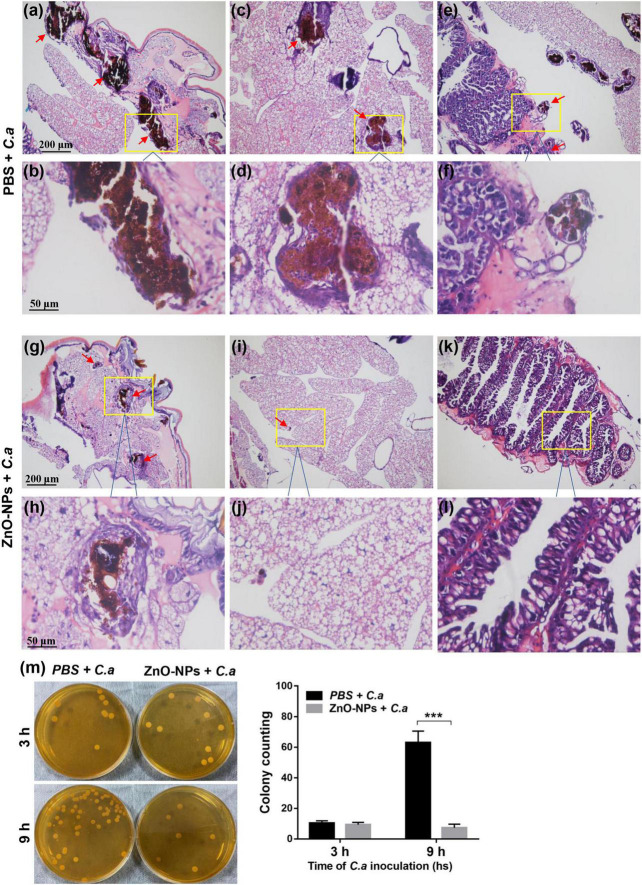
Histopathological analysis and determination of fungal burden in *G. mellonella*. **(a–l)** Representative photomicrographs of the fungal morphology, distribution, and burden in *G. mellonella* on day 2 after ZnO-NP pretreatment and *C. albicans* inoculation. *G. mellonella* pretreated with PBS and inoculated with *C. albicans* was used as the control. Stained sections are shown at ×100 magnification **(a,c,e,g,i,k)** and ×400 magnification **(b,d,f,h,j,l)**, respectively. *C. albicans* were in yeast morphological form and incorporated in melanized nodules among tissues. The strain isolates were visualized in subcuticular areas **(a,b,g,h)**, fat bodies **(c,d,i,j)**, and intestinal walls **(e,f)**. Smaller nodules were observed in ZnO-NP-pretreated *G. mellonella* **(g–j)** than in PBS-pretreated control group **(a–f)**. **(k,l)** No fungus was detected in the intestines of ZnO-NP-pretreated *G. mellonella*. **(m)** The fungal burden in the hemolymph of PBS or ZnO-NP-pretreated *G. mellonella* after challenge with *C. albicans* (1 × 10^5^ CFU/larva). At 3 and 9 h postinfection, hemolymph was obtained from 15 replicates per group and was diluted 10 times with PBS. Then 100 μl of diluted hemolymph was plated onto yeast peptone dextrose (YPD) solid medium and incubated at 37°C for 24 h. Colony counts were measured and shown as mean ± SD. Differences were determined by using the *t*-test. ***p < 0.001.

The fungal morphology, distribution, and burden in larval tissue were studied by histopathological analysis 48 h after being challenged with *C. albicans*. The strain cells on the H&E staining section were easily distinguished as brown in bright field optics. Figures 3a–j showed that *C. albicans* was in yeast morphological form and incorporated in melanized nodules among the tissues. In the ZnO-NP-pretreated larvae, the strain cells developed smaller nodules localized in the subcuticular area (Figures 3a,b,g,h) and fat body (Figures 3c,d,i,j) compared with those in the PBS-pretreated controls. Some fungal elements reached the intestinal wall (Figures 3e,f) in PBS-pretreated larvae, while no fungus was detected in the intestines of ZnO-NP-pretreated larvae (Figures 3k,l). Hence, the administration of ZnO-NPs effectively reduced *C. albicans* dissemination in the larval tissue.

Hemolymph collected from infected larvae pretreated with ZnO-NPs or PBS was spread on YPD plates to quantify the fungal burden. As shown in Figure 3m, 3 h after *C. albicans* infection, no significant difference in fungal load was observed between ZnO-NPs and PBS-pretreated larvae (9.33 ± 0.88 *vs.* 10.33 ± 0.88 CFU, *p* = 0.46). Interestingly, 9 h after infection, the fungal load statistically decreased in the ZnO-NP-pretreated larvae but increased in the PBS control group (7.33 ± 1.33 *vs.* 63.00 ± 4.36 CFU, *p* = 0.0003), which demonstrated that ZnO-NPs contribute to the killing of fungus within larvae. Particularly, 9 h rather than 3 h of inoculation of ZnO-NPs triggered a noticeable antifungal effect. Accordingly, it can be inferred that it takes a longer time for ZnO-NPs to exert *in vivo* antifungal activity, indicating that this antifungal effect might not be a direct effect.

### Zinc Oxide Nanoparticles Possessed No Direct Antifungal Capability Against *Candida albicans in vitro*

**FIGURE 4:**
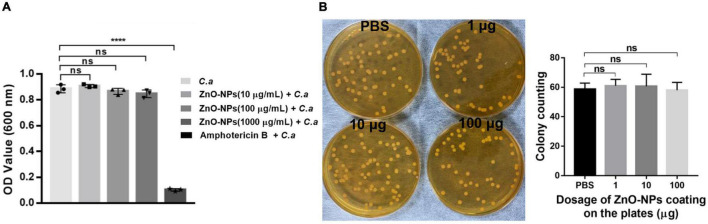
The direct antifungal capability of ZnO-NPs against *C. albicans in vitro*. **(A)** The growth of *C. albicans* in YPD broth containing different concentrations of ZnO-NPs (0–1,000 μg/ml) after overnight incubation. *C. albicans* incubated with amphotericin B (0.5 μg/ml) were used as the positive control. *C. albicans* incubated in the absence of ZnO-NPs and amphotericin B were used as the negative control. The minimum inhibitory concentration (MIC) value was assessed by measuring absorbance at 600 nm. **(B)** The growth of *C. albicans* on YPD plates coated with ZnO-NPs. One hundred microliters of PBS solutions containing 1, 10, and 100 μg of ZnO-NPs were evenly spread on YPD plates followed by plating with approximately 60 CFU of *C. albicans* and incubation at 37°C for 24 h. The same volume of PBS was coated as a control. Colonies were captured and counted for assessing the fungal growth. Each experiment was repeated in triplicate independently. Data are shown as mean ± SD. The differences in the mean values between groups were assessed using one-way analysis of variance (ANOVA) method.

To figure out whether the saving of infected larvae by ZnO-NPs was due to the direct antifungal capability of the nanomaterials, *in vitro* antifungal activity of ZnO-NPs against *C. albicans* was first evaluated by liquid broth assay. *C. albicans* was cultured in YPD broth containing different concentrations of ZnO-NPs (0–1,000 μg/ml) at 37°C for 24 h. As shown in Figure 4A, 0.5 μg/ml of amphotericin B was utilized as positive control. However, no significant growth inhibition was observed in the ZnO-NP-treated groups. It could be speculated that ZnO-NPs did not inhibit the growth of *C. albicans* directly at the tested concentrations.

Moreover, the growth of *C. albicans* was assessed on YPD plates coated with 1, 10, and 100 μg of ZnO-NPs on the agar surfaces. As shown in Figure 4B, there was no significant difference in CFU of *C. albicans* between ZnO-NPs and PBS-coated plates. Meanwhile, we did not observe any apparent discrepancy in the colony diameter of all groups, indicating no growth inhibition. These results confirmed that ZnO-NPs did not possess fungicidal effects at the tested concentrations. Hence, we concluded that the protective role of ZnO-NPs in *G. mellonella* against *C. albicans* infection did not depend on their fungicidal capability.

### Zinc Oxide Nanoparticles Increased the Larval Hemocyte Density

We continued to investigate the ability of ZnO-NPs in enhancing the innate immune responses of *G. mellonella*. We quantified the major parameters of the larval immune response, including hemocyte density and aggregation, phagocytosis ability of hemocyte, and phenoloxidase activity of hemolymph.

**FIGURE 5:**
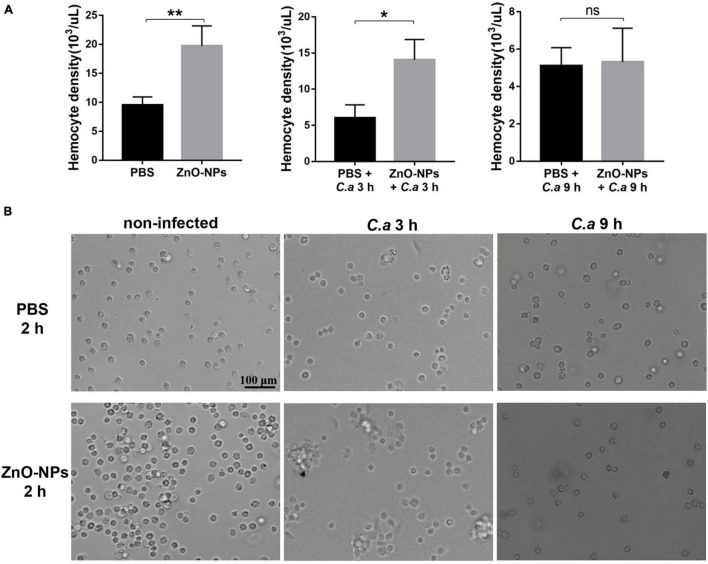
The density and aggregation of hemocytes in ZnO-NP-pretreated *G. mellonella* with/without *C. albicans* challenge. **(A)** Quantification of hemocytes in ZnO-NP-pretreated *G. mellonella* with/without *C. albicans* infection. Differences were determined by using the t-test. *p < 0.05; **p < 0.01. **(B)** Representative photomicrographs showing the aggregation of hemocytes in ZnO-NP-pretreated *G. mellonella* with/without *C. albicans* infection. Hemocytes formed small aggregates in response to the administration of ZnO-NPs in the absence of *C. albicans* infection. After 3-h exposure to *C. albicans*, intense aggregation of hemocytes was observed in ZnO-NP-pretreated *G. mellonella*. Scale bar, 100 μm.

First, the hemocyte density in ZnO-NPs-treated *G. mellonella* was measured in the absence of *C. albicans* infection. As indicated in Figure 5A (left), the increased hemocyte density in the larvae 2 h post-ZnO-NP treatment, with a recording of 19.73 ± 1.95 × 10^3^ cells/μl, significantly differed from those of 9.52 ± 0.81 × 10^3^ cells/μl in the PBS-treated larvae (*p* = 0.0091). Second, we measured the larval hemocyte density in ZnO-NP-pretreated *G. mellonella* at the indicated time postinfection of *C. albicans* (Figure 5A). Following infection for 3 h, the larvae pretreated with ZnO-NPs presented increased hemocyte density (14.07 ± 1.62 × 10^3^ cells/μl) compared with the larvae pretreated with PBS as control (6.04 ± 1.03 × 10^3^ cells/μl, *p* = 0.014). Furthermore, 9 h postinfection, there was no significant difference in hemocyte density between the two groups (5.11 ± 0.56 × 10^3^ cells/μl *vs.* 5.32 ± 1.04 × 10^3^ cells/μl, *p* = 0.87).

As for the decrease in hemocyte density after 9 h of infection, it may be related to the consumption of hemocytes in nodulation or encapsulation process (Kavanagh and Reeves, 2004). Therefore, we speculated that ZnO-NP treatment increased the hemocyte density of larvae, contributing to defending against *C. albicans*.

### Zinc Oxide Nanoparticles Promoted the Aggregation of Larval Hemocytes

The aggregation of hemocytes in larvae is one of their innate immune responses, which was observed under microscopy in this study. Hemocytes in hemolymph were extracted from the 2 h-pretreated larvae for microscopy. As shown in Figure 5B, hemocytes formed small aggregates in response to the administration of ZnO-NPs. No hemocyte aggregates were found in the control group injected with PBS.

Furthermore, combined with 3 h exposure to *C. albicans*, intense aggregation of hemocytes was observed in the larvae pretreated with ZnO-NPs, presenting as nodule formation, while cells in the control group were still distributed separately (Figure 5B). With the extension of infection time to 9 h, we noticed a reduction in large nodules in the ZnO-NP-pretreated group, in which the aggregation of hemocytes was similar to that of the control group.

Based on the above results, it was concluded that ZnO-NPs could increase the hemocyte density and induce the formation of microaggregates, resulting in a rapid immune response to invasive pathogens, which presented as intense aggregation around the pathogen.

### Micromorphology Changes of Hemocytes *in Galleria mellonella*

**FIGURE 6:**
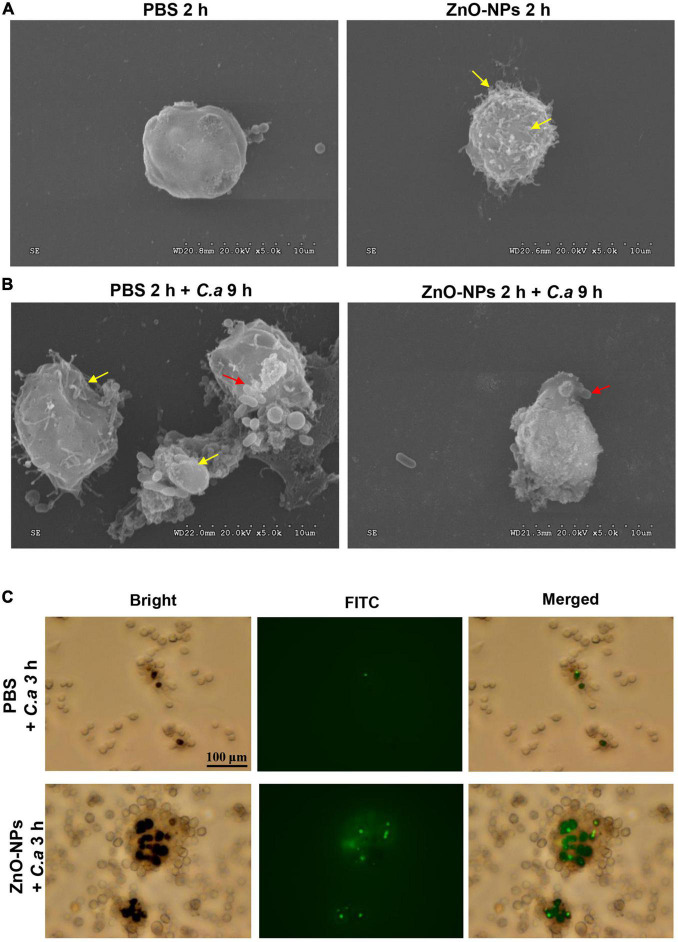
Micromorphology change and phagocytosis of hemocytes in ZnO-NP-pretreated *G. mellonella* with/without *C. albicans* challenge. **(A,B)** Scanning electron microscopy (SEM) presenting micromorphology change of hemocytes collected from ZnO-NP-pretreated *G. mellonella* with/without *C. albicans* challenge. PBS pretreatment was applied as the control. **(A)** The yellow arrows indicate shrinking and burring protrusion on the surface of hemocytes exposed to ZnO-NPs without *C. albicans* infection. **(B)** Following the challenge with *C. albicans* for 9 h, hemocytes presented irregular and partly destroyed by *C. albicans* (yellow arrows) in PBS-pretreated larvae but hemocytes engulfed *C. albicans* (red arrow) significant morphological changes in ZnO-NP-pretreated larvae. Micromorphology change was shown at ×5,000 magnification. **(C)** Representative microphotographs showing phagocytosis of fluorescein isothiocyanate isomer (FITC)-labeled *C. albicans* by hemocytes in *G. mellonella*. Larvae were pretreated with ZnO-NPs or PBS and then challenged with FITC-labeled *C. albicans* for 3 h at 37°C. Hemolymph was obtained from each larva separately and 10-fold diluted in a 12-well plate for microscope observation. Scale bar, 100 μm.

Scanning electron microscopy was used for capturing the micromorphology of hemocytes in the ZnO-NP-pretreated larvae with/without *C. albicans* challenge. First, Figure 6A presents the differences in morphology hemocytes collected from the larvae treated with ZnO-NPs or PBS in the absence of infection. Specifically, hemocytes from ZnO-NPs-treated larvae shrunk and had some burring protrusion on the surface, indicating cell activation. Following inoculation with *C. albicans* for 9 h, the phagocytosis of *C. albicans* by hemocytes was captured and is shown in Figure 6B. With PBS pretreatment, hemocytes presented irregularly and were partly destroyed by *C. albicans*. With ZnO-NP pretreatment, the phagocytosis showed more favorably, without significant morphological changes in hemocytes.

### Zinc Oxide Nanoparticles Enhanced the Phagocytosis of Larval Hemocytes on *Candida albicans*

Furthermore, we utilized fluorescent microscopy to assess the phagocytosis of larval hemocytes against *C. albicans*. The fungus cells were labeled with FITC and injected into ZnO-NP-pretreated larvae. As shown in Figure 6C, 3 h postinfection, hemocytes in the larvae pretreated with ZnO-NPs were more susceptible and aggressive to wrap up *C. albicans* than cells in the PBS-pretreated larvae.

### Zinc Oxide Nanoparticles Activated Phenoloxidase Activity of Larval Hemolymph

The activation of phenoloxidase in the hemolymph is critical due to the involvement in immune defense and was examined with/without *C. albicans* infection in the pretreated larvae in the current study. The phenoloxidase enzyme activity was quantified by OD_490_ value with a microplate reader.

**FIGURE 7:**
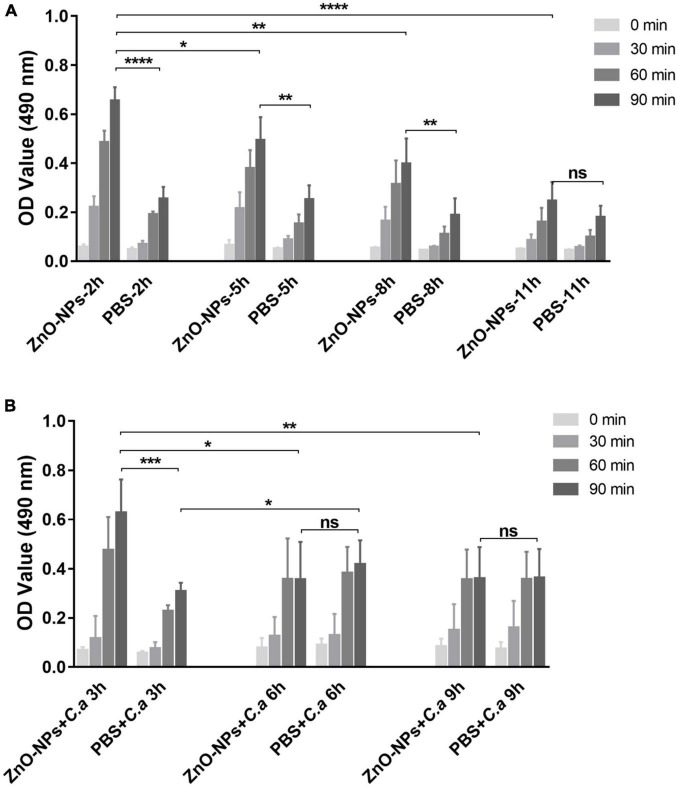
Phenoloxidase (PO) activity in the hemolymph of ZnO-NP-pretreated *G. mellonella* with/without *C. albicans* challenge. **(A)** The PO activity in the hemolymph of ZnO-NP-treated *G. mellonella* at indicated time points after the inoculation. The same volume of PBS was injected as the control. **(B)** The PO activity in the hemolymph of *G. mellonella* receiving ZnO-NP pretreatment and *C. albicans* infection. Larvae 2 h post-ZnO-NPs or PBS treatment were immunized with *C. albicans* (1 × 10^5^ CFU cells per larva) and used for detection of PO activity at 3, 6, and 9 h after challenge. The PO activity was determined using DOPA as a substrate based on melanin formation by measuring absorbance at 490 nm. The enzyme activity was quantified over 90 min at 15-min intervals presented as mean ± SD. The kinetics of the PO activity values were calculated from a representative experiment performed in triplicate. Difference analysis was assessed by using one-way analysis of variance (ANOVA) methods. *p < 0.05; **p < 0.01; ***p < 0.001; ****p < 0.0001.

First, larvae were treated with ZnO-NPs or PBS only in the absence of infection. As shown in Figure 7A, hemolymph was extracted 2–11 h after the ZnO-NP injection, while PBS treatment was utilized as the control. At the indicated time points, the phenoloxidase activity was monitored over 90 min at 15-min intervals. In the ZnO-NP-injected larvae, the phenoloxidase activity remained at a higher level than in the PBS-injected controls (Figure 7A). The phenoloxidase activity peaked 2 h postinoculation of ZnO-NPs, presenting 2.57-fold higher activity than that of PBS-inoculated control larvae (0.654 ± 0.057 vs. 0.254 ± 0.049, *p* < 0.0001). We could tell from Figure 7A that phenoloxidase activity showed a trend of decrease according to the prolongation of ZnO-NP treatment from 2 to 11 h. Until 11 h postinjection, the phenoloxidase enzyme activity was equal in ZnO-NP and PBS-treated larvae (0.246 ± 0.077 *vs.* 0.213 ± 0.058, *p* = 0.43; Figure 7A). Thus, according to the results, the administration of ZnO-NPs activated the phenoloxidase system. Moreover, the activation of larval phenoloxidase took place in a short time after ZnO-NP inoculation and then became attenuated over time lapse.

Second, we detected the phenoloxidase activity levels in ZnO-NPs and PBS-pretreated larvae 3, 6, and 9 h postchallenge with *C. albicans*. As shown in Figure 7B, 3 h postinfection, the larvae pretreated with ZnO-NPs exhibited 2.04-fold higher phenoloxidase activity than the PBS-pretreated controls (0.627 ± 0.136 *vs.* 0.307 ± 0.036, *p* = 0.0009). The activation of phenoloxidase 2 h post-inoculation of ZnO-NPs may contribute to the enhanced immune response defending against the subsequent *C. albicans* infection. As the infection time extended to 6 h, the phenoloxidase activity reduced in the ZnO-NP-pretreated larvae (0.355 ± 0.154) but increased in the PBS-pretreated ones (0.417 ± 0.098). There was no significant difference in the phenoloxidase activity of the groups at either 6 or 9 h after infection. The activation of the phenoloxidase system by ZnO-NPs in *G. mellonella* at the early stage probably plays a role in defending against the subsequent infections.

## Discussion

During the last decade, lots of NPs have been reported as novel and sustainable antifungal materials (Staron and Dlugosz, 2021). A previous work has revealed that ZnO-NPs have a wide antifungal range and relatively low toxicity (Sharma et al., 2020). However, the data of ZnO-NPs were primarily obtained from *in vitro* assays. Thus, we attempted to figure out the *in vivo* toxicity and antifungal activity of ZnO-NPs. *Galleria mellonella* larvae have been widely used as an animal model to study the pathogenesis of microorganisms and assess the antimicrobial activity of new compounds (Frei et al., 2021). In addition, some research extended the utilization of larvae to study NPs (Moya-Anderico et al., 2021a,b). Given that *C. albicans* is one of the most common fungal pathogens and has increased resistance to traditional antifungal agents, we established a *G. mellonella*–*C. albicans* infection model to elucidate the antifungal capacity of ZnO-NPs. We herein got an essential finding that ZnO-NPs contribute to saving larvae from *C. albicans* infection at low doses without noticeable toxicity. Intriguingly, ZnO-NPs protected the larvae against fungal infection by inducing the innate immune responses rather than directly killing the fungus *in vivo*. An understanding of the mechanisms driving the antimicrobial ability of ZnO-NPs is important for proposing new strategies to eliminate the burden of *C. albicans* and to prevent host death.

A nanoparticle refers to a microscopic particle with a size less than 100 nm in one or more external dimensions (Lavker et al., 2021). The ZnO-NPs used in this study are nanorod shaped and with an average size of 100 nm × 15 nm. The shape and size of ZnO-NPs reported in the literature were diverse, such as spherical shape with a size of 7.41–13 nm (David et al., 2021), crystallite shape with a size of 5–50 nm (Saleh et al., 2021), and rod shape with a size of 88 nm (Ebadi et al., 2021). The size and shape of NPs play an essential role in determining their toxicity (Sukhanova et al., 2018). For example, NPs larger than 10 nm are less toxic than NPs in sizes of several nanometers because larger NPs are not easy to enter the nucleus and interact with DNA (Huo et al., 2014). Mesoporous silica NPs in a size of 100 nm were effectively adsorbed on the surface of the erythrocyte without disturbing the membrane or morphology (Zhao et al., 2011). In addition, *in vitro* experiment on cultured BEAS-2B cells has shown that needle- and plate-shaped NPs produced more potent cytotoxicity than sphere and rod-shaped NPs (Zhao et al., 2013). In the present study, we evaluated the toxicity of ZnO-NPs *in G. mellonella* larvae by intra-hemocoel injection and determined the LD50 and LD99 values as 400 and 800 mg/kg, respectively. A previous study has reported that the LD50 and LD99 of 70-nm ZnO rod-shaped NPs for force-fed larvae were 6.03 and 12.86 μg/10 μl, respectively, equivalent to 83 and 176 mg/kg body weight (Eskin et al., 2019). The inconsistent results of the two studies might be due to the difference in the size of ZnO-NPs, the modes of administration, the larval size and age, and culture conditions.

In this work, ZnO-NP pretreatment saved larvae from *C. albicans* infection at a dose of 2 mg/kg without apparent toxicity, which is manifested by reducing the larval mortality and the fungal burden in hemolymph and tissue. The mechanisms driving antimicrobial activity of ZnO-NPs have been described in the literature, including generation of ROS, the release of zinc ions, and interaction of ZnO-NPs with the surfaces of microorganisms (Behzad et al., 2021; Efthimiou et al., 2021). Particularly, these mechanisms mainly focus on the directly antimicrobial properties of NPs. Intriguingly, we found that ZnO-NPs at the tested concentrations did not inhibit the growth of *C. albicans*. Similarly, reported by Visnapuu et al. (2018), in dark conditions, the ZnO-covered surface did not exhibit a significant antimicrobial effect for *C. albicans*, while under UVA illumination, ZnO was slightly toxic to *C. albicans*. Moreover, *C. albicans* was significantly less sensitive to ZnO-covered surface compared with *Escherichia coli* and *Staphylococcus aureus* (Visnapuu et al., 2018). The failure of antimicrobial action in our study is probably due to the low level of released Zn^2+^ ions from ZnO-NPs in the dark and the insensitivity of *C. albican*s to Zn^2+^ ions.

Since no direct antifungal activity of ZnO-NPs was observed in our study, we assume that the life extension of infected larvae by ZnO-NPs might be mainly attributed to impacting the immune defense in hosts. As we know, the immune system of *G. mellonella* larvae has structural and functional similarities to the innate immune response in mammals, which provides possibilities of utilizing it to study the interaction between hosts and pathogens (Tsai et al., 2020). Considering that hemocytes play a crucial role in cellular immune response as they serve as phagocytic cells, the induced reaction of hemocytes by ZnO-NPs was verified in larvae. Moreover, since phenoloxidase-catalyzed melanization is a key humoral immune response in larvae, the phenoloxidase enzyme activity in larval hemolymph was evaluated. The cellular and humoral immune defense were activated by ZnO-NP pretreatment, evidencing increased hemocyte density, aggregation, phagocytosis ability, and phenoloxidase activity. Additionally, in ZnO-NP-treated larvae, the reduced destruction of hemocytes was evident from the SEM micrographs, especially in cells that phagocytized *C. albicans*. The above evidence indicates that ZnO-NP protect *G. mellonella* against *C. albicans* infection *via* immune activation in the host instead of direct fungicidal actions.

The abovementioned mode of ZnO-NPs on the immune response was similar to the immune priming effect in *G. mellonella*. “Immune priming” is an enhanced innate immune response that enables invertebrates to withstand potentially lethal infections (Sheehan et al., 2020). This process can be triggered by microbial or components of microbial cells, antifungal drugs, or thermal and physical stress, indicating a generalized antimicrobial response (Sheehan et al., 2020). Our work revealed that the introduction of ZnO-NPs into the hemocoel of larvae increased the hemocyte density, even in a short term of 2 h. Hemocytes probably migrated from a sessile population normally present under the larval cuticle rather than *de novo* synthesis (Kavanagh and Reeves, 2004; Sheehan et al., 2020). Besides, we noticed distinct aggregation of hemocytes and enhanced phagocytosis in response to ZnO-NP injection. These results were in agreement with previous research conducted *in vitro* that nano-ZnO films could promote microbial clearance by macrophages and polymorphonuclear leukocytes by facilitating phagocytic efficacy (Rutherford et al., 2021). It has been recently revealed that the phenoloxidase activity of *G. mellonella* was induced after *C. albicans* infection (Sheehan and Kavanagh, 2018). In our study, both ZnO-NPs and PBS-pretreated larvae exhibited increased phenoloxidase activity after *C. albicans* challenge. However, ZnO-NP-pretreated larvae exhibited a higher peak of phenoloxidase activity at the earlier stage than PBS-pretreated control, implying a stronger immune defense against subsequent infections. Based on our results and preceding studies, it could be concluded that ZnO-NP-induced hemocyte aggregation and phagocytosis as well as phenoloxidase activation in the hemolymph was probably conducive to pathogen clearance quickly and effectively after *C. albicans* challenge.

## Conclusion

In general, we first evaluated the *in vivo* toxicity of ZnO-NPs on *G. mellonella* larvae and found the enhanced immune response. The relatively low concentrations of ZnO-NPs did not display any evident toxicity; however, it primed a protective immune response in the larvae against sublethal fungal infection. These results showed that the low toxicity properties and immunomodulatory potentials of ZnO-NPs make it particularly attractive in medical applications. Especially, the ZnO-NP-based immunomodulator is a promising tool to defend against fungal infections. Future research is worth delineating the molecular mechanisms of immunomodulatory effects mediated by ZnO-NPs and whether the ZnO-NP-induced immune activation could be extended to other NPs.

## Data Availability Statement

The raw data supporting the conclusions of this article will be made available by the authors, without undue reservation.

## Author Contributions

KZ and X-WH conceived the study. M-NX, WP, H-XZ, and X-MP performed the research. S-QD, Y-MT, and LL analyzed the data. X-WH and M-NX drafted the manuscript. LL and WP edited the manuscript. All authors have read and approved the final manuscript.

## Conflict of Interest

The authors declare that the research was conducted in the absence of any commercial or financial relationships that could be construed as a potential conflict of interest.

## Publisher’s Note

All claims expressed in this article are solely those of the authors and do not necessarily represent those of their affiliated organizations, or those of the publisher, the editors and the reviewers. Any product that may be evaluated in this article, or claim that may be made by its manufacturer, is not guaranteed or endorsed by the publisher.
